# Dynamic lineage evolution in hematologic malignancies: a case report of B-ALL Progressing to CMML and AML

**DOI:** 10.1007/s00277-026-07132-8

**Published:** 2026-06-15

**Authors:** Sheikh Abdullah, Mariana Marrero Castillo, Shiva Jashwanth Gaddam, Benedict Amalraj, Faryal Shoaib, Poornima Ramadas

**Affiliations:** 1 Department of Internal Medicine, LSU Health Sciences Center Shreveport, 1501 Kings Highway, Shreveport, LA 71103 USA; 2 Department of Hematology and Oncology, LSU Health Sciences Center Shreveport, 1501 Kings Highway, Shreveport, LA 71103 USA; 3 Department of Pathology, Louisiana State University Health Sciences Center, Shreveport, LA USA

**Keywords:** Lineage Switch, B-cell acute lymphoblastic leukemia, Chronic myelomonocytic leukemia, Acute myeloid leukemia, KRAS mutation, Clonal evolution

## To the Editor,

**Fig. 1:**
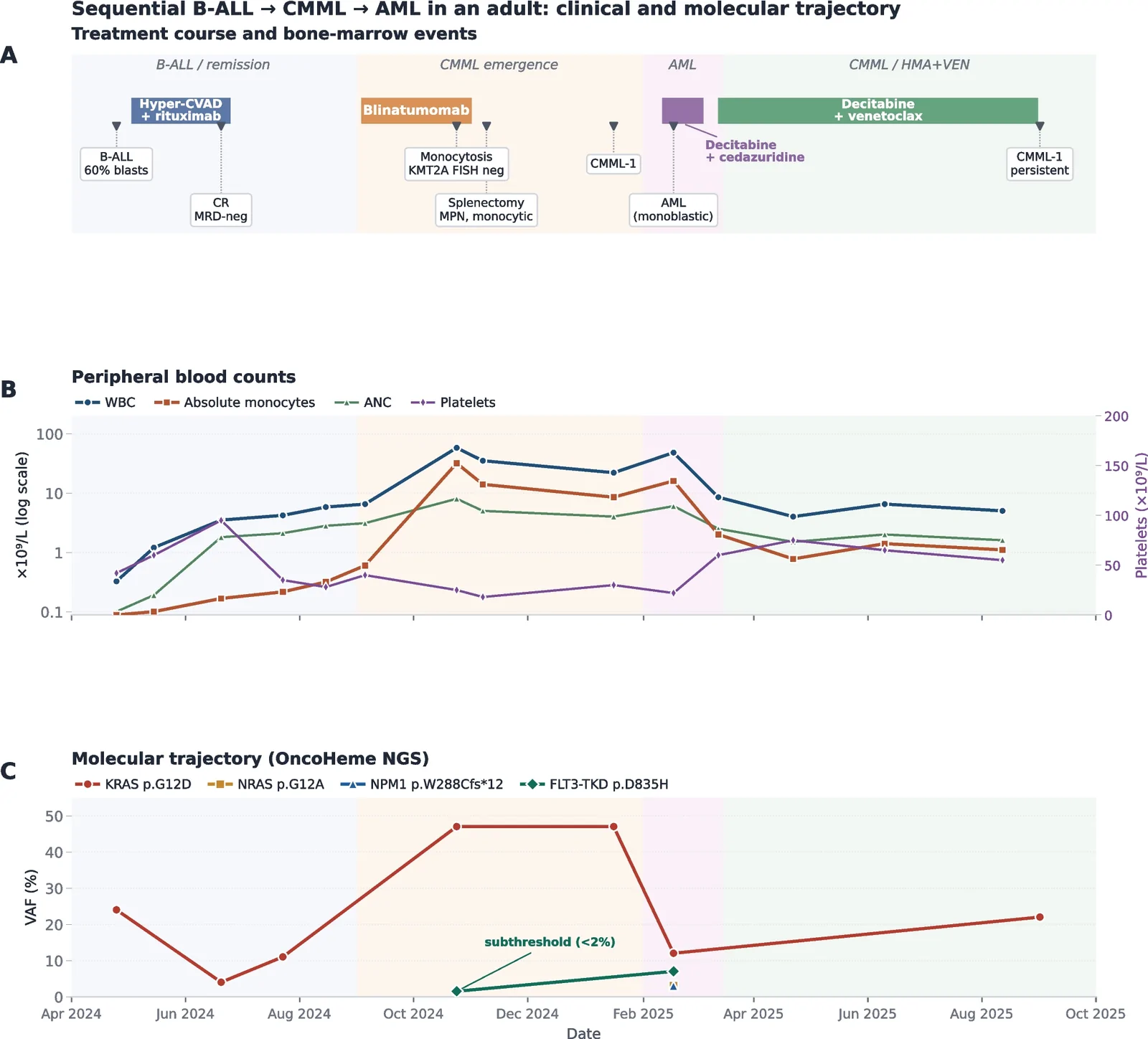
Clinical and molecular trajectory of sequential B-ALL → CMML → AML in an adult. (**A**) Treatment course (Hyper-CVAD with rituximab; blinatumomab; oral decitabine/cedazuridine [Inqovi]; decitabine plus venetoclax) and key bone-marrow events from April 2024 through August 2025. (**B**) Peripheral-blood counts on a symmetric-log scale for white-blood-cell count (WBC), absolute neutrophil count (ANC), and absolute monocyte count, with platelet count on the right axis. (**C**) Variant allele frequency (VAF) trajectories from serial OncoHeme NGS panels. *KRAS* p.G12D persisted at 4–11% during morphologic and clonoSEQ-MRD–negative remission, rose to 47% as CMML emerged, declined with cytoreduction, and tracked with disease activity. *NRAS* p.G12A, *NPM1* type-A (p.Trp288Cysfs*12), and *FLT3*-TKD p.D835H emerged at AML transition (the *FLT3*-TKD variant had been detected sub-threshold during the CMML-emergence phase). *KMT2A* break-apart FISH was negative; *TP53* was wild-type at all timepoints. HMA + VEN = hypomethylating agent plus venetoclax

Lineage switching is an increasingly recognized mode of relapse in B-cell acute lymphoblastic leukemia (B-ALL), most often manifesting as transition to acute myeloid leukemia (AML) and enriched in cases harboring *KMT2A* rearrangement and following CD19-directed immunotherapy [1, 2]. In Project EVOLVE, an international cohort of post-immunotherapy lineage-switch cases, 70.7% of switches were B-ALL to AML or mixed-phenotype acute leukemia, 64% harbored *KMT2A* rearrangements, and the median overall survival after switch was 4.8 months [3]. Adult cases are particularly aggressive [4, 5], and direct B-ALL-to-monocytic-AML transitions have been described [6]; however, sequential evolution through a defined intermediate myeloid neoplasm is rarely documented in adults [7]. We describe a 69-year-old woman whose Philadelphia-negative B-ALL evolved through chronic myelomonocytic leukemia (CMML) to acute monoblastic leukemia, with serial molecular profiling supporting a bilineal RAS-driven neoplasm rather than a strict single-clone lineage switch (Fig. 1).

**Fig. 2:**
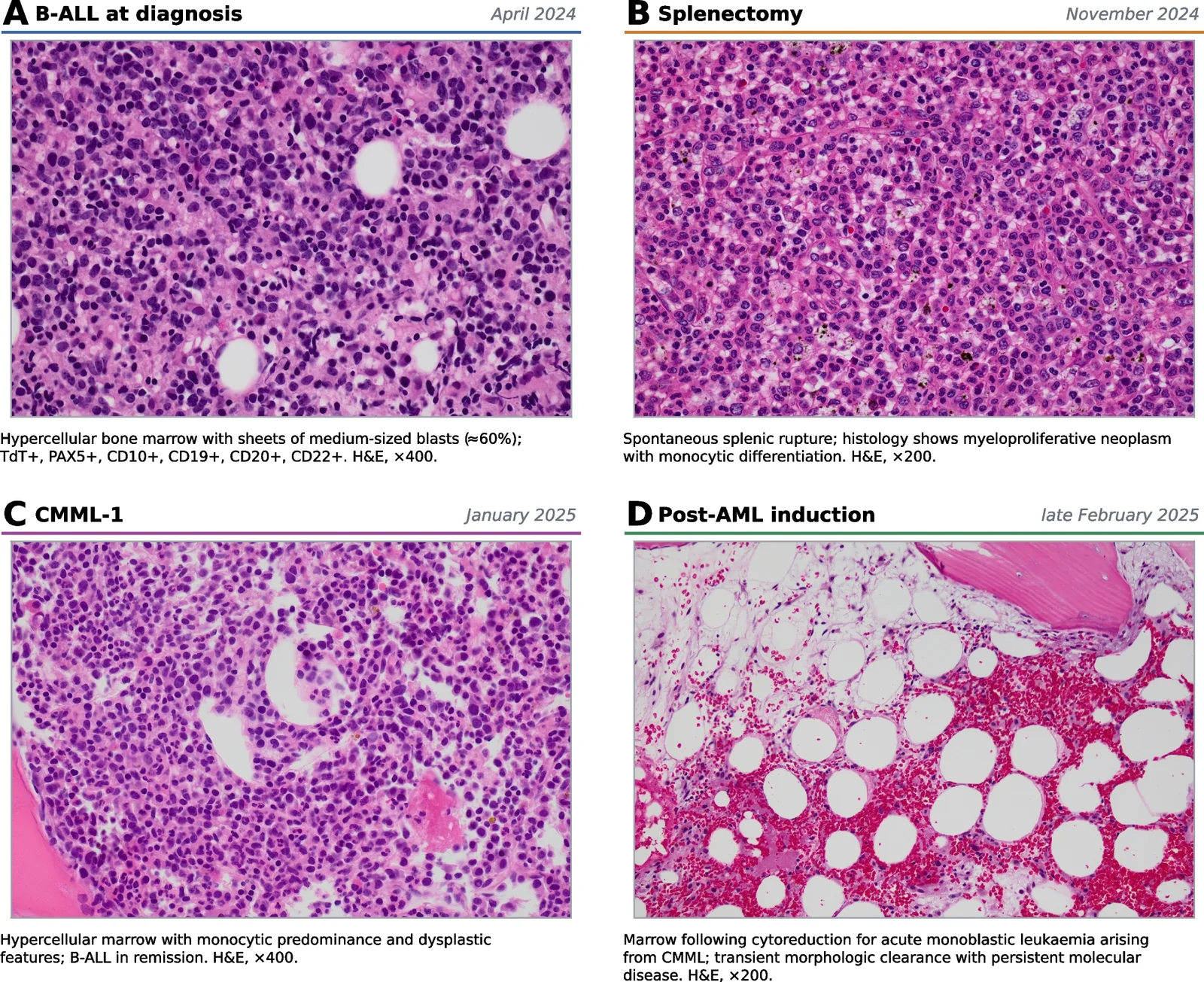
Composite histopathology and immunophenotypic findings illustrating the lineage trajectory. (**A**) B-ALL at diagnosis (April 2024): hypercellular bone marrow with sheets of medium-sized blasts (≈60%), positive for TdT, PAX5, CD10, CD19, CD20, and CD22 by immunohistochemistry/flow cytometry (H&E, × 400). (**B**) Splenectomy (November 2024) following spontaneous splenic rupture: histology shows a myeloproliferative neoplasm with monocytic differentiation (H&E, × 200). (**C**) Bone-marrow biopsy demonstrating CMML-1 (January 2025): hypercellular marrow with monocytic predominance and dysplastic features; B-ALL remained in remission (H&E, × 400). (**D**) Post-induction bone-marrow biopsy (late February 2025) following cytoreduction for acute monoblastic leukemia arising from CMML, showing transient morphologic clearance with persistent molecular disease (H&E, × 200)

A 69-year-old woman with hypertension and hyperlipidemia presented in April 2024 with profound pancytopenia (representative WBC 0.56 × 10^9^/L; absolute neutrophil count [ANC] 0.10 × 10^9^/L; hemoglobin 7.2 g/dL; platelets 42 × 10^9^/L). Bone-marrow biopsy showed approximately 60% blasts that were TdT + , PAX5 + , CD10 + , CD19 + , CD20 + , and CD22 + , consistent with B-ALL (Fig. 2A). Residual non-blast hematopoiesis was largely effaced by the blast infiltrate, and overt dysplastic features were not appreciated; peripheral-blood morphology was not suggestive of an underlying myelodysplastic neoplasm (MDS) at presentation. Cross-sectional imaging at presentation (CT abdomen/pelvis, MRI abdomen, MRI brain) demonstrated a benign hepatic hemangioma and an incidental 1 cm meningioma along the left planum sphenoidale, with no evidence of solid-organ malignancy. Cytogenetics demonstrated hyperdiploidy (trisomies 3, 6, 12, 14, 18, 21 in 7/20 metaphases). The ALL FISH panel was *BCR–ABL1*–negative; an *IKZF1* partial deletion was present in 10% of nuclei, and three copies of the *ETV6*, *RUNX1*, and *IGH* regions were observed in 20% of nuclei. Targeted myeloid next-generation sequencing (NGS; Mayo Clinic OncoHeme 47-gene panel; sensitivity ≥ 2–4% variant allele frequency [VAF]) detected *KRAS* c.35G > A (p.G12D) at 24% VAF; no pathogenic variants were identified in any of the other 46 genes on the panel, including *TP53*. clonoSEQ (IgH-based MRD) was sent.

She received Hyper-CVAD with rituximab and intrathecal methotrexate. Bone-marrow examinations in June and July 2024 confirmed morphologic and clonoSEQ-MRD–negative remission, with decreased megakaryocyte numbers; low-level *KRAS* p.G12D persisted (4–11% VAF). She developed steroid-responsive leukocytoclastic-vasculitis-like skin lesions (direct immunofluorescence negative). Persistent thrombocytopenia prompted deferral of cycle 2B; she transitioned to blinatumomab (28 μg/24 h) in September 2024. By October 2024 she developed marked monocytosis (WBC 57.84 × 10^9^/L; monocytes 55%; absolute monocyte count > 1 × 10^9^/L sustained on serial counts and on flow cytometry over more than three months). Bone marrow showed ongoing B-ALL remission with monocytic expansion; overt morphologic dysplasia was not appreciated at this timepoint, and peripheral-blood smear showed monocytic predominance without circulating blasts. ALL-FISH and AML-FISH were normal, including a normal *KMT2A* break-apart pattern (*nuc ish KMT2A(MLL)* × *2* [200/200 cells]), with no evidence of rearrangement or copy-number gain, and *KRAS* p.G12D rose to 47% VAF, while *TP53* remained wild-type. Repeat cross-sectional imaging during the leukocytoclastic vasculitis (LCV) episode and at the time of monocytosis demonstrated no solid-organ malignancy.

In November 2024 she developed disseminated intravascular coagulation, splenomegaly, and spontaneous splenic rupture, a recognized but rare complication of acute leukemias [8], requiring embolization and subsequent splenectomy; the splenic histology demonstrated a myeloproliferative neoplasm with monocytic differentiation (Fig. 2B). A repeat bone-marrow biopsy in January 2025 was consistent with CMML-1 (Fig. 2C; *KRAS* p.G12D 47% VAF; clonoSEQ-MRD negative; *TP53* wild-type). Despite oral decitabine/cedazuridine, peripheral-blood flow cytometry in February 2025 demonstrated 56% dim-CD45 blasts (CD10 partial, CD11b + , CD13 dim, CD14 + , CD16 + , CD33 + , CD34 partial, CD56 + , CD64 + , HLA-DR +) with a second 28% monocytoid population, most consistent with acute monoblastic leukemia arising from CMML. NGS detected *KRAS* p.G12D (12%), *NPM1* type-A (p.Trp288Cysfs*12, 3%), and *NRAS* p.G12A (3%); a low-level *FLT3*-TKD p.D835H (~ 7%; previously detected at sub-threshold level) was also identified. *TP53* remained wild-type, and 17p was uninvolved on FISH. A persistent *TET2* c.3662G > A variant of uncertain significance was noted across timepoints (2–8% VAF), without acquisition of canonical CMML co-mutations such as *ASXL1* or *SETBP1* [9]. She received decitabine plus venetoclax. Her course was complicated by recurrent CMML-1 (*NPM1*-negative on flow August 2025; Fig. 2D shows the post-AML-induction marrow), transfusion dependence, and recurrent vasculitic-appearing lesions; she was subsequently lost to follow-up. The integrated clinical and molecular trajectory is shown in Fig. 1.

Several features of this case warrant comment. Lineage evolution proceeded sequentially through a defined CMML phase before AML transformation; most reported adult lineage-switch cases transition directly from B-ALL to AML or mixed-phenotype acute leukemia within 1–6 months of CD19-directed therapy [3, 4, 6], and only one prior adult case has been documented passing through an intermediate myeloid neoplasm, in that case an intervening myelodysplastic neoplasm rather than CMML [7]. The absence of *KMT2A* rearrangement (excluded by FISH break-apart probe) and the wild-type *TP53* status distinguish this case from the typical post-immunotherapy lineage-switch literature [3] and from *KMT2A*-amplified, *TP53*-mutant cases such as that of Takeda and colleagues [10]. CMML-1 was diagnosed by sustained absolute monocytosis > 1 × 10^9^/L for more than three months in conjunction with characteristic bone-marrow morphology, monocytic differentiation on splenectomy histology, and a clonal myeloid mutational profile, in keeping with the WHO 5th-edition criteria [11]. Reactive causes of monocytosis were considered but could not account for the magnitude, duration, splenic involvement, or persistent clonal mutation profile.

The strongest molecular argument for shared clonal ancestry is the persistence and dynamic VAF trajectory of *KRAS* p.G12D: detectable at 4–11% during morphologic and clonoSEQ-negative remission, rising to 47% as CMML emerged, declining to 12% with cytoreduction, and tracking with disease activity through AML therapy (Fig. 1C). This pattern is most parsimoniously interpreted as a bilineal RAS-driven neoplasm rather than a strict single-clone lineage switch: an initial *KRAS* p.G12D-mutant progenitor gave rise to a dominant lymphoid B-ALL clone and a quieter myeloid clone that, lacking canonical CMML co-mutations such as *ASXL1* or *SETBP1* [9], remained clinically silent during the B-ALL-dominant phase and expanded only when therapy selectively suppressed the lymphoid clone. Under this framework, the case does not require, and indeed does not have, the canonical drivers of a B-ALL-to-AML lineage switch (*KMT2A* rearrangement, *TP53* mutation), because it represents two parallel clones differing in initial tempo. We acknowledge that *KRAS* p.G12D is a recurrent hotspot in both B-ALL and myeloid neoplasms, and that convergent evolution cannot be formally excluded without paired whole-exome or whole-genome sequencing demonstrating shared private (non-hotspot) variants [2]; *KMT2A*-partial tandem duplication cannot be excluded by the OncoHeme panel without RNA-seq or whole-genome sequencing, and bi-allelic *TP53* inactivation by larger 17p alterations cannot be excluded by an SNV/indel panel alone, although both FISH panels were uninvolved at 17p.

The temporal relationship between blinatumomab and the emergence of the myeloid clone is best interpreted as a “rebound” expansion of a pre-existing, RAS-driven myeloid population once the dominant lymphoid clone was selectively suppressed. Cytopenias, vasculitis, and decreased megakaryocyte numbers were already present before blinatumomab was initiated, and the interval from the last Hyper-CVAD cycle through the first blinatumomab cycle is sufficient for an occult myeloid clone to expand into the cytoreductive niche; such rebound expansion is well recognized during salvage therapy of transformed CMML, particularly in proliferative variants. Prior cytotoxic chemotherapy (Hyper-CVAD with rituximab and cyclophosphamide) provided the initial cytoreductive opening [12], while the lymphoid-targeted effect of blinatumomab plausibly lifted competitive constraints on the parallel myeloid clone, paving the way for its propagation. Most blinatumomab-associated lineage switches reported to date have occurred in *KMT2A*-rearranged disease [3, 13], which our patient lacked, supporting a role for blinatumomab as a selective enabler of an already-established RAS-mutant myeloid clone rather than a direct driver of lineage commitment.

This case has implications for surveillance and rechallenge strategies. IgH-based MRD assays such as clonoSEQ cannot detect emerging myeloid clones, a self-evident but clinically consequential limitation that was illustrated repeatedly in our patient. Lineage-agnostic MRD strategies that may have allowed earlier detection of the parallel myeloid clone include error-corrected NGS targeting persistent somatic mutations such as *KRAS* (or quantitative *NPM1* MRD once acquired) [14], multi-lineage flow-cytometry panels capable of identifying monocytic and myeloid-blast populations during ALL remission, and emerging cell-free DNA approaches. Persistent cytopenias, unexplained monocytosis, or new immune phenomena during morphologic ALL remission should prompt comprehensive bone-marrow re-evaluation including myeloid-focused NGS and AML/CMML FISH (with *KMT2A* break-apart and copy-number assessment), and consideration of fusion-detection methodologies if *KMT2A*-PTD or rare structural variants remain clinically suspect. Recent work also indicates that the lineage commitment of switched leukemias may be plastic, raising the possibility of selective rechallenge with CD19-directed therapy in carefully selected cases when a B-lineage compartment re-emerges [15], although such rechallenge would not have been informative in our patient, whose disease evolved into a stable myeloid phenotype without B-lineage re-emergence.

In summary, this case extends the spectrum of bilineal RAS-driven hematologic malignancies in adults. A shared *KRAS* p.G12D founder mutation generated two coexisting clones, an initially dominant Philadelphia-negative B-ALL and an indolent myeloid clone, that were unmasked sequentially by therapy: the myeloid clone propagated as clinically apparent CMML-1 once the lymphoid clone was selectively suppressed by CD19-directed therapy, and subsequently transformed to acute monoblastic leukemia with acquisition of *NPM1*, *NRAS*, and low-level *FLT3*-TKD lesions. The absence of *KMT2A* rearrangement and *TP53* mutation distinguishes this case from the prototypical post-immunotherapy lineage-switch literature and supports a bilineal-clone model over a strict lineage-switch interpretation. Comprehensive baseline molecular profiling and lineage-agnostic minimal residual disease surveillance are essential to anticipate and characterize these uncommon but aggressive relapses.

## Data Availability

No datasets were generated or analysed during the current study.
